# Activation of the IL-2 Receptor in Podocytes: A Potential Mechanism for Podocyte Injury in Idiopathic Nephrotic Syndrome?

**DOI:** 10.1371/journal.pone.0157907

**Published:** 2016-07-07

**Authors:** Arnold H. Zea, Tyrus Stewart, Jeannine Ascani, David J. Tate, Beatriz Finkel-Jimenez, Anna Wilk, Krzysztof Reiss, William E. Smoyer, Diego H. Aviles

**Affiliations:** 1 Department of Microbiology, Immunology, and Parasitology, Louisiana State University Health Sciences Center, New Orleans, Louisiana, United States of America; 2 Department of Pediatric Nephrology, Louisiana State University Health Sciences Center, New Orleans, Louisiana, United States of America; 3 Department of Research Ochsner Biobank, Ochsner Health System, New Orleans, Louisiana, United States of America; 4 Eurofins Central Analytical Laboratories, New Orleans, Louisiana, United States of America; 5 Department of Medical Microbiology and Immunology, American University of the Caribbean School of Medicine, Coral Gables, Florida, United States of America; 6 Department of Genetics, Louisiana State University Health Sciences Center, New Orleans, Louisiana, United States of America; 7 Department of Nephrology, Nationwide Children’s Hospital, Columbus, Ohio, United States of America; Max-Delbrück Center for Molecular Medicine (MDC), GERMANY

## Abstract

The renal podocyte plays an important role in maintaining the structural integrity of the glomerular basement membrane. We have previously reported that patients with idiopathic nephrotic syndrome (INS) have increased IL-2 production. We hypothesized that podocytes express an IL-2 receptor (IL-2R) and signaling through this receptor can result in podocyte injury. To confirm the presence of the IL-2R, we tested a conditionally immortalized murine podocyte cell line by flow cytometry, qPCR, and Western blot. To test for the presence of the IL-2R in vivo, immunohistochemical staining was performed on human renal biopsies in children with FSGS and control. Podocytes were stimulated with IL-2 in vitro, to study signaling events via the JAK/STAT pathway. The results showed that stimulation with IL-2 resulted in increased mRNA and protein expression of STAT 5a, phosphorylated STAT 5, JAK 3, and phosphorylated JAK 3. We then investigated for signs of cellular injury and the data showed that pro-apoptotic markers Bax and cFLIP were significantly increased following IL-2 exposure, whereas LC3 II was decreased. Furthermore, mitochondrial depolarization and apoptosis were both significantly increased following activation of the IL-2R. We used a paracellular permeability assay to monitor the structural integrity of a podocyte monolayer following IL-2 exposure. The results showed that podocytes exposed to IL-2 have increased albumin leakage across the monolayer. We conclude that murine podocytes express the IL-2R, and that activation through the IL-2R results in podocyte injury.

## Introduction

Idiopathic nephrotic syndrome (INS) is a clinical condition occurring mainly in children. It is characterized by massive proteinuria, hypoalbuminemia, hyperlipidemia and edema. The most common histologic diagnoses in patients with INS are minimal change nephrotic syndrome (MCNS) and focal segmental glomerulosclerosis (FSGS) [1]. Most patients with MCNS remain steroid responsive with a good long term outcome. However, 20% of these patients develop steroid resistant idiopathic nephrotic syndrome (SRINS) with progression to end-stage renal disease (ESRD) [2]. In up to 50% of these cases, nephrotic syndrome recurs almost immediately after renal transplantation, suggesting the existence of a circulating factor responsible for nephrotic syndrome in these patients [3].

The podocyte is a cell that plays a key role in INS. Defects to podocyte-specific genes nephrin and podocin can cause nephrotic syndrome [4–6]. However, it is important to note that single gene mutations account for only a minority of patients with INS. In patients with INS, it has been postulated that cytokines produced by T-cells increase the permeability of the glomerular basement membrane [7–9].

Potential permeability factors in INS include: vascular permeability factor (VPF), hemopexin and soluble urokinase receptor (suPAR) [10–12]. The binding of suPAR to its receptor in podocytes results in foot process fusion and proteinuria [12]. However recent studies do not support the role of suPAR in differentiating between patients with idiopathic FSGS and those with secondary FSGS, or patients with FSGS and other glomerular diseases [13–17].

Serum from patients with INS during relapse, have increased levels of cytokines such as IL-2, IL-4, and IL-8 [18–20]. We have reported that T-lymphocytes from patients with INS in relapse (MCNS, FSGS) have increased IL-2 mRNA [21]. Furthermore, it has been demonstrated in cancer patients, that treatment with IL-2 can induce proteinuria which resolves once this therapy is discontinued [22]. Infusion of IL-2 into rats in vivo results in podocyte foot process fusion and proteinuria [23]. Despite this clear association, the mechanism for IL-2-induced proteinuria remains unknown.

Autophagy, a regulated lysosomal pathway that plays a role in the recycling of the cytoplasm and the elimination of non-functional organelles, is crucial for the survival and homeostasis of cells [24]. Podocytes maintain a high basal level of autophagy and defective autophagy could play a role in facilitating podocyte injury [25]. Inhibition of autophagy could induce podocyte apoptosis by activation the pro-apoptotic pathway of the endoplasmic reticulum [26]. In the current study, we demonstrated the expression of IL-2R in murine podocytes and demonstrated the effect of IL-2 on podocyte apoptosis and autophagy. We hypothesize that murine podocytes express a functional IL-2R, which upon activation may cause injury to the podocyte.

## Methods

### Podocyte culture and stimulation

The conditionally immortalized murine podocyte clone JR07 was cultured as previously described [27]. Briefly, cells were cultured in RPMI 1640 medium containing 10% fetal bovine serum (Hyclone, Logan, UT, USA), 100 U/mL penicillin, 100 μg/mL streptomycin, and 0.292 mg/ml glutamine (Gibco-BRL, Rockville, MD, USA). Podocyte cultures were expanded by growth in medium containing 10 U/mL mouse interferon-γ (INF-γ) (Sigma Chemical Co., St. Louis, MO, USA) at 33°C with 100% relative humidity and 5% CO_2_ atmosphere, and were induced to differentiate by culture at 37°C in medium without INF-γ. Podocytes were allowed to differentiate for 10 days with media changes every 3 days. Titration experiments were performed to determine the concentration of IL-2 for our model. The concentration of 100ng/ml resulted in optimal activation of the IL-2R. Prior to harvest at day 10, cells were stimulated with 100 ng/ml of IL-2 (Innovative Research, Novi, MI). For the IL-2 receptor studies, we isolated RNA and protein after incubation with IL-2 for 21 hours. In the experiments for JAK/STAT signaling, we isolated RNA and protein in controls and stimulated cells at 30 and 60 minutes following the stimulation with IL-2. For the experiments with mitochondrial potentials and apoptosis, podocytes were stimulated with IL-2 (100ng/ml) for 72hrs to determine the presence of apoptosis or mitochondrial depolarization. The podocytes were incubated with IL-2 for 72 hrs for the albumin influx assays. The protein expression of LC3, cFLIP and Bax was also measured after 72 hrs stimulation.

### Flow Cytometry

After 10 days of differentiation, cells were trypsinized and rinsed with PBS. Five hundred thousand cells were stained with 4μg of the following markers: IL-2Rα (CD25), IL-2Rβ (CD122), IL-2Rγ (CD132) (BD Biosciences, San Jose, CA) for 15 min at 4°C. Cells were washed with PBS twice at 1000 x g at 4°C. After the final rinse step, cells were fixed with 2% paraformaldehyde in PBS. Simultest γ_1_/γ_2a_ (Beckman Coulter) was used as an isotype control and treated the same as the other cell markers. The samples were read on a FACScalibur (Beckton and Dickinson) at the LSUHSC-Cell Analysis and Immunology core.

### qRT-PCR

Podocytes were extracted directly into PureZOL RNA isolation reagent (Bio-Rad, Hercules, CA) and processed using the Aurum total RNA isolation kit, leaving out the DNase treatment. Following elution of the RNA from the spin columns, samples were submitted to DNase treatment using Turbo DNA-free (Ambion, Austin, TX). RNA concentration was measured by absorbance at 260 nm. Quantitative RT-PCR was performed on a My iQ Real Time PCR Detection System using the iScript One-Step RT-PCR kit with SYBR Green (Bio-Rad, Hercules, CA). All reactions were performed with the following conditions: 5 min denaturation at 95°C followed by 40 cycles of 95°C for 10 seconds and 55°C for 30 seconds and a final annealing step. After amplification, a melt curve was obtained for all reactions to determine that a single amplicon existed for each sample with each primer set. All results were measured as fold-induction compared to GAPDH signal in the same sample. Primer sets are listed in Table 1.

**Table 1:** Quantitative Reverse Transcriptase Primers.

Gene	Forward primer	Reverse primer	Source
IL-2Rα	ATTTGTCATGGGAGTTGCTGGTGC	TGCCACATTCAAAGCCCTCTCCTA	NM_008367
IL-2Rβ	ACAGCTGTCCTCAAGTTGTGATGT	TCAGGACCTCTTCGTTTGGGTTGT	NM_008368.4
IL-2Rγ	ATTTCGGGTTCGGAGCCGCTTAA	ACAGGGATAAGCACAGCTTCCAGT	NM_013563
Jak3	GCTTCCACACAATTCCAACGGACA	CATCAAGCTTGCGGCTTCCAGAAA	NM_008413
STAT5a	TGTGCCCTCAACCTCACTACAACA	TCATCCAGGTCAAACTCGCCATCT	NM_001164062
STAT5b	GCTGTGTGAAGCGCTCAACATGAA	AGGACACGGACATGCTGTTGTAGT	NM_001113563.1
GAPDH	TCAACAGCAACTCCCACTCTTCCA	ACCCTGTTGCTGTAGCCGTATTCA	NM_008084

Accession numbers from NCBI database; primers designed using Primer Quest program from IDT DNA

### Western blot

Twenty-five micrograms of protein extracts were solubilized in Laemmli buffer and β-mercaptoethanol followed by electrophoresis on NuPAGE Bis-Tris gels (Life Technologies, Grand Island, NY). Protein was then transferred to polyvinylidene difluoride (PVDF) membranes (Life Technologies, Grand Island, NY) and blocked in 5% milk for 1hr. Antibodies used are listed in Table 2. Membranes were then incubated with the appropriate HRP conjugated secondary antibody. Bands were visualized by chemiluminescence (SuperSignal West Femto Maximum Sensitivity Substrate, Thermo Scientific, Waltham, MA). All bands were normalized to the corresponding β-actin or GAPDH band (Sigma, St. Louis, MO).

**Table 2:** Antibody list for western blot.

Antibody	Company	Catalog number	Concentration
IL-2Rα	Santa Cruz	sc-666	1:200
IL-2Rα	BD Biosciences	557425	1:500
IL-2Rβ	Santa Cruz	sc-16427	1:500
IL-2Rγ	BD Biosciences	554455	1:500
STAT5a	Santa Cruz	sc-136081	1:200
pSTAT5	Santa Cruz	sc-11761	1:1,000
Jak3	Santa Cruz	sc-6932	1:1,000
pJak3	Santa Cruz	sc-16567	1:1,000
Bax	Santa Cruz	sc-493	1:5,000
cFlip_L_	Santa Cruz	sc-8346	1:1,000
LC3	Novus Biologicals	NB100-2220	1:5,000
Podocin	Santa Cruz	sc-21009	1:1,000
Nephrin	Santa Cruz	sc-377246	1:500
β-actin	Sigma Aldrich	A2228	1:10,000

### Immunohistochemistry

Paraffin-embedded tissue from renal biopsies of children was analyzed. Sections 4-μm thick were deparaffinized, washed in distilled water and finally equilibrated in 10 mM citric acid, pH 6.0, for 10 min. For antigen retrieval slides were baked in an oven for 45min at 60°C. After the slides were allowed to cool down to room temperature they were washed with phosphate-buffered saline (PBS) and incubated in blocking solution (3% H_2_O_2_ in methanol) for 10 min. The sections were then incubated in 2.5% horse serum for 20min followed by primary antibody (Anti-human/rabbit IL2-Ralpha) diluted in 2.5% horse serum overnight at 4°C. Slides were then washed in PBS and incubated with appropriate secondary antibody for 30min at room temperature. Slides were washed in PBST, then fresh DAB solution was applied for 1min. Slides were then rinsed in distilled water and counterstained with hematoxylin.

### Mitochondrial Membrane Potential (ΔΨm) Assay

ΔΨm and apoptosis were measured by the flow cytometry based Guava EasyCyte MitoPotential assay (Guava Technologies). Loss of ΔΨm is measured by a cationic dye (JC-1) that fluoresces either green or orange depending upon ΔΨm. 7-AAD was used in conjunction with JC-1 to measure apoptosis. Following 72hrs of IL-2 stimulation, cells were harvested by trypsinization and stained with JC-1 and 7-AAD for 30min. The cells were then immediately analyzed by Guava EasyCyte flow cytometer using Mito-Potential software.

### Albumin Influx Assay

An albumin infiltration assay was used to monitor the integrity of a podocyte monolayer following treatment with or without IL-2. Murine podocytes (JR07) were seeded onto transwell filters (3μm pore, Corning) at (5–6 X 10^3^ cells/well) and allowed to differentiate for 10 days. Following this differentiation period, the cells were serum starved and treated with or without IL-2 (100ng/ml, R&D Systems) for 72hrs. After 72hrs of treatment, the medium was removed and the cells were washed twice with PBS supplemented with 1 mmol/L MgCl_2_ and 1 mmol/L CaCl_2_ to preserve cadherin based junctions. The transwell insert was then filled with pre-warmed (37°C) 0.15 ml of RPMI 1640 and the bottom well filled with pre-warmed (37°C) 1ml of RPMI supplemented with 40 mg/ml BSA. The cells were then incubated at 37°C and small aliquots of media (100μl) were taken at various time points. Albumin concentration was measured in the sample aliquots by bicinchoninic acid assay kit (Pierce) according to manufacturer’s instructions.

### Statistical Analysis

Intergroup comparisons were performed by unpaired t-test or one-way analysis of variance using Graph pad Prism 4 (Graph Pad Software Inc., San Diego, California).

P≤ 0.05 was considered to be significant. Tukey’s multiple comparisons test was performed with ANOVA for comparison between groups.

## Results

### Expression of the IL-2 Receptor in murine podocytes

**Fig 1:**
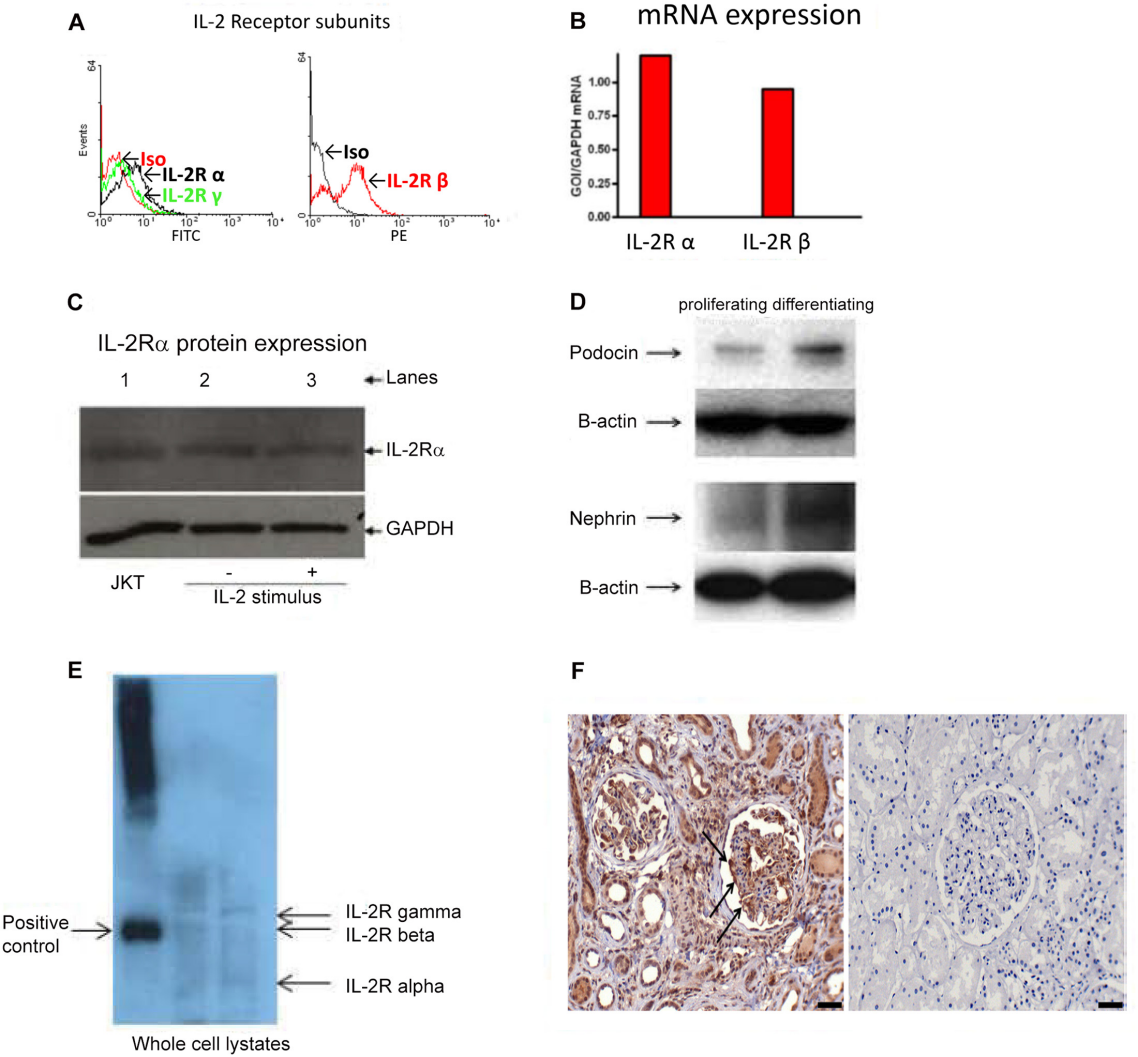
IL-2R subunits are present in differentiated murine podocytes. (A) Representative flow cytometry histograms showing surface IL-2R subunits α and γ (black and green histograms). Nonspecific staining (black histogram) was determined using an isotype-specific control antibody. Murine podocytes express the three subunits of the IL-2R. (B) IL-2R α alpha and β chains are expressed in non-stimulated podocytes. Differentiated podocytes express α alpha and β mRNA subunits of the IL-2R as shown by real time PCR. (C) The alpha subunit is demonstrated by Western blot. The positive control consists of protein extracts obtained from Jurkat (JKT) cells. (D) The conditionally immortalized murine podocyte clone JR07 expresses podocyte specific proteins nephrin and podocin in proliferating and differentiating cells as demonstrated by Western blot. (E) Whole cell lysates of murine podocytes were immunoblotted with anti- alpha, beta, and gamma chains antibodies showing the presence of the whole complex of the IL-2R by Western blot. (F) IL-2R expression in podocytes from human renal biopsies. Immunohistochemical staining was performed on human renal biopsies from children with FSGS and control. CD25 (alpha chain) expression was observed in biopsies from children with FSGS on the surface podocytes, indicated by the arrows (left panel) when compared to the biopsy control (right panel).

**Fig 2:**
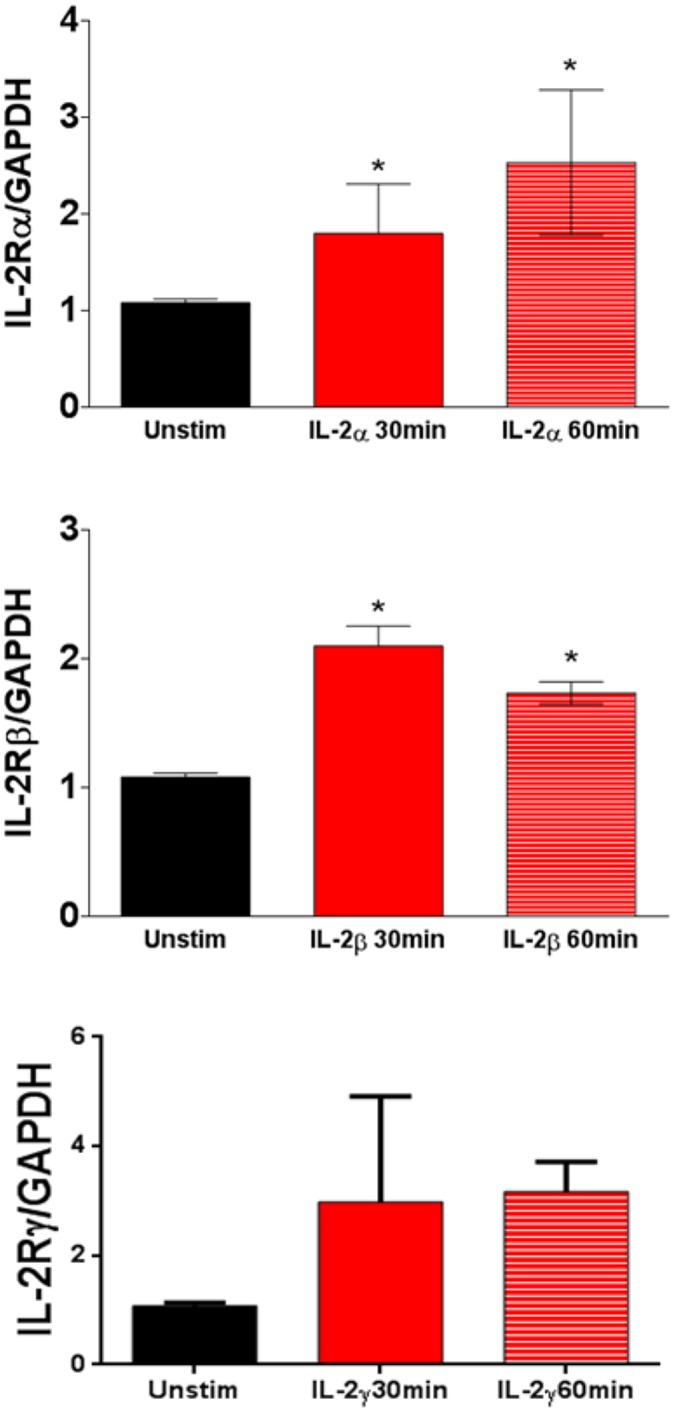
Stimulation with IL-2 increases mRNA expression of all three subunits of the IL-2R in differentiated murine podocytes. All the subunits were detected by qRT-PCR within 30 minutes after stimulation with IL-2. There are significant increases in the expression of the three subunits when normalized to GAPDH (P< 0.05). N = 3 in individual groups. P<0.05 (One-way ANOVA). Bar columns and error bars represent mean and standard deviation respectively.

Although the IL-2R is expressed in kidney tubular epithelial cells, there are no previous studies demonstrating the presence of IL-2R in murine podocytes [28]. Initial experiments were pursued to establish the presence of the IL-2R in unstimulated murine podocytes. We detected the expression of the three subunits of the IL-2R by flow cytometry (Fig 1A). The gene and protein expression of the IL-2Rα was also confirmed by qPCR and Western blot (Fig 1B and 1C). Podocytes in culture were verified by expression of podocyte specific proteins nephrin and podocin (Fig 1D). Expression of the alpha, beta, and gamma chains of the IL-2R complex were demonstrated by Western blot (Fig 1E), and the expression of the IL2R-alpha was demonstrated by Immunohistochemistry of stained human renal biopsies, (Fig 1F).Next, we measured gene expression of the IL-2R subunits following 30 and 60 minutes of IL-2 stimulation by real-time PCR using primer pairs specific for subunits of IL-2R. The results show that after 30 minutes of IL-2 stimulation the podocytes have significantly increased gene expression of IL-2R subunits, this increase was ultimately sustained after 60 minutes of stimulation (Fig 2). These results demonstrate that murine podocytes express the IL-2R and show that the IL-2R is functional.

### IL-2R signaling in podocytes

**Fig 3:**
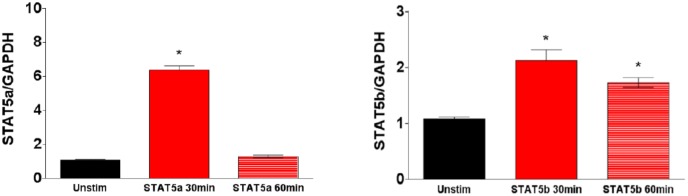
Stimulation with IL-2 increases mRNA expression of STAT 5 subunits. Expression of STAT 5a-b subunits in non-stimulated podocytes were compared to podocytes stimulated with IL-2. The mRNA expression of STAT 5a-b was significantly increased after stimulation with IL-2 (P< 0.01, P<0.05 respectively; One-way ANOVA). N = 3 in individual groups. Bar columns and error bars represent mean and standard deviation respectively.

**Fig 4:**
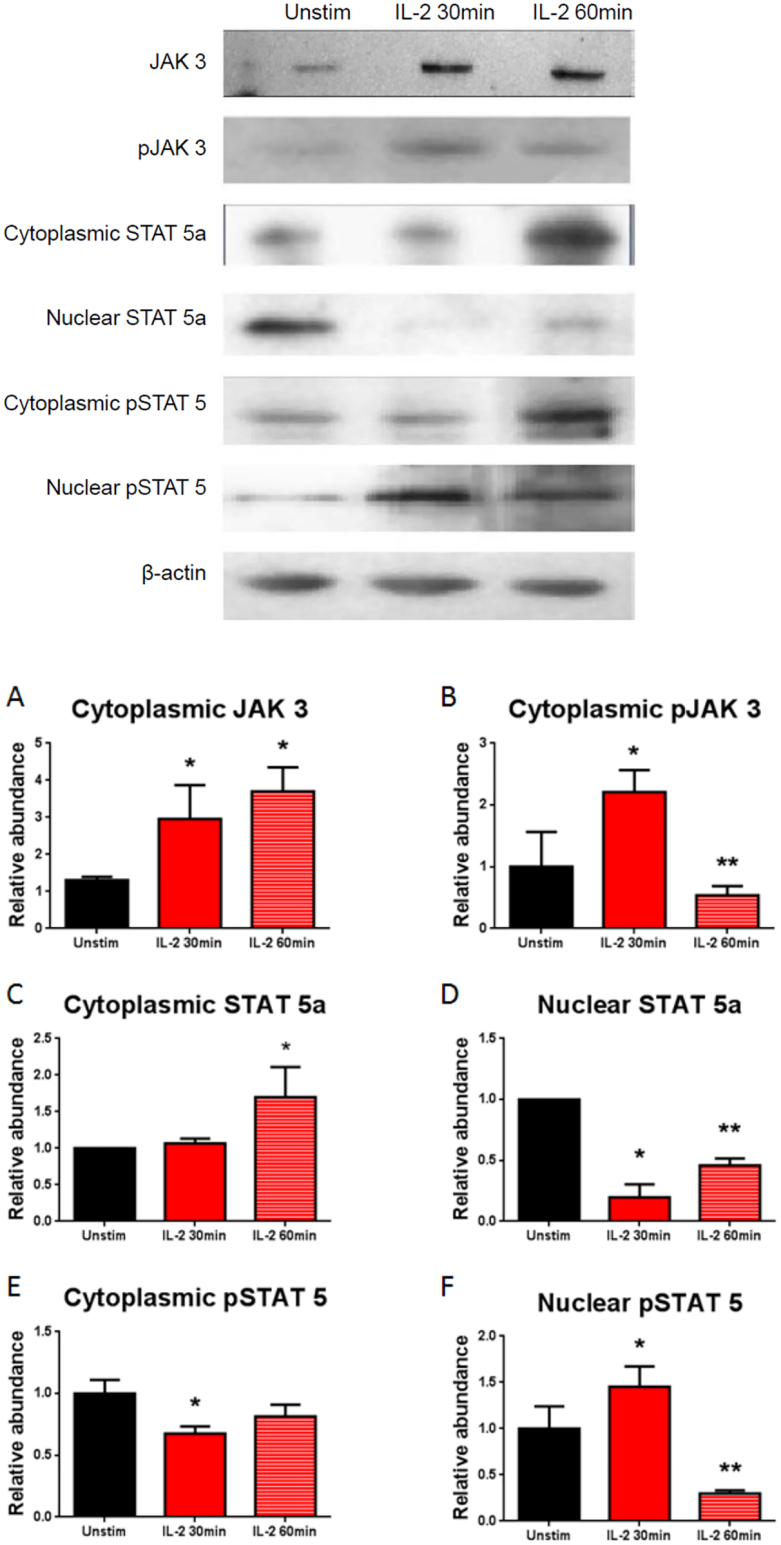
Activation of the IL-2 receptor is associated with induction of JAK/STAT protein expression. Expression of JAK 3, pJAK 3, STAT 5a, and pSTAT 5 in non-stimulated podocytes were compared to podocytes stimulated with IL-2. (A)There is increased expression of JAK 3 after both 30 and 60 minutes of IL-2 stimulation (P<0.05, One-way ANOVA with Tukey’s multiple comparisons test). N = 3 in individual groups. (B) Phosphorylated JAK 3 is significantly increased after 30 minutes of stimulation with IL-2 (*, P<0.01); however, after 60 minutes the expression has returned to within unstimulated levels (**, P<0.01; One-way ANOVA with Tukey’s multiple comparisons test). N = 3 in individual groups. (C) Cytoplasmic STAT 5a protein expression is significantly increased in podocytes after 60 minutes of IL-2 stimulation (P<0.0001, One-way ANOVA with Tukey’s multiple comparisons test) compared to unstimulated podocytes. N = 3 in individual groups. (D) Nuclear STAT 5a protein expression in podocytes was significantly decreased at both time points with IL-2 stimulation (*, P<0.0001) compared to unstimulated podocytes. However, podocytes stimulated for 60 minutes showed a significantly higher STAT 5a protein expression when compared to 30 min (**, P<0.0001; One-way ANOVA with Tukey’s multiple comparisons test). N = 3 in individual groups. (E) Cytoplasmic phosphorylated STAT 5 protein expression was significantly decreased following 30 minutes of stimulation with IL-2, but this effect was not sustained after 60 minutes of stimulation (P<0.05, One-way ANOVA with Tukey’s multiple comparisons test). N = 3 in individual groups. (F) Similar to phosphorylated JAK3, phosphorylated nuclear STAT 5 protein expression was significantly increased after 30 minutes of IL-2 stimulation, but was significantly decreased after 60min of IL-2 stimulation compared to both unstimulated podocytes and podocytes stimulated with IL-2 for 30 minutes (Fig 4F). (P<0.001, One-way ANOVA with Tukey’s multiple comparisons test). N = 3 for individual groups. Bar columns and error bars represent mean and standard deviation respectively.

The JAK-STAT pathway plays an integral role in IL-2-driven activation in lymphoid cells [29]. Therefore, we evaluated the expression of the JAK-STAT pathway in IL-2 stimulated and non-stimulated podocytes by real-time PCR and Western blot. Samples were collected following 30 and 60 minutes of stimulation with IL-2. The stimulated podocytes showed increased gene expression of both STAT 5a and STAT 5b by real-time PCR (Fig 3). The results showed that JAK 3 protein expression was significantly increased following 30 minutes of IL-2 stimulation and was sustained after 60 minutes (Fig 4A). Similarly, phosphorylated JAK 3 was also significantly increased after 30 minutes of IL-2 stimulation; however, after 60 minutes of IL-2 stimulation the protein expression had significantly decreased (Fig 4B). Cytoplasmic STAT 5a protein expression was also significantly increased after 60 minutes of IL-2 stimulation (Fig 4C). Nuclear STAT 5a protein expression was significantly decreased following IL-2 stimulation (Fig 4D). Cytoplasmic phosphorylated STAT 5 was significantly decreased after 30 minutes of IL-2 stimulation but returned to unstimulated level after 60 minutes of stimulation (Fig 4E). Similar to phosphorylated JAK3, phosphorylated nuclear STAT 5 protein expression was significantly increased after 30 minutes of IL-2 stimulation, but was significantly decreased after 60min of IL-2 stimulation compared to both unstimulated podocytes and podocytes stimulated with IL-2 for 30 minutes (Fig 4F).

### IL-2 induces podocyte apoptosis

**Fig 5:**
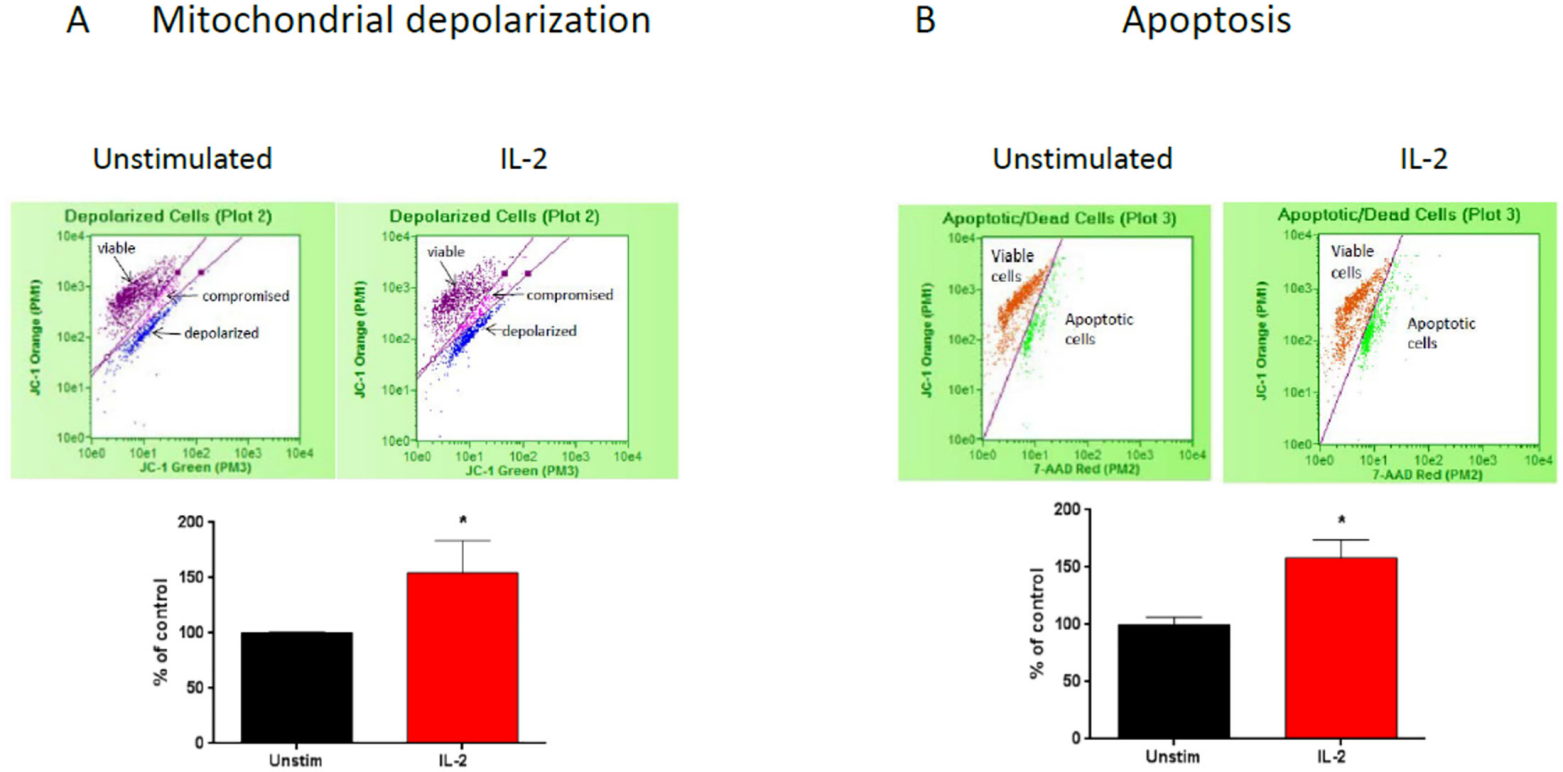
IL-2 induces podocyte mitochondrial depolarization and apoptosis. Flow cytometry measured mitochondrial membrane potentials and apoptosis in podocytes. (A) IL-2 induces a significant increase in mitochondrial membrane depolarization. (B) Incubation with IL-2 also resulted in increased apoptosis (P< 0.05, unpaired t-test). N = 4 for individual groups. Bar columns and error bars represent mean and standard deviation respectively.

**Fig 6:**
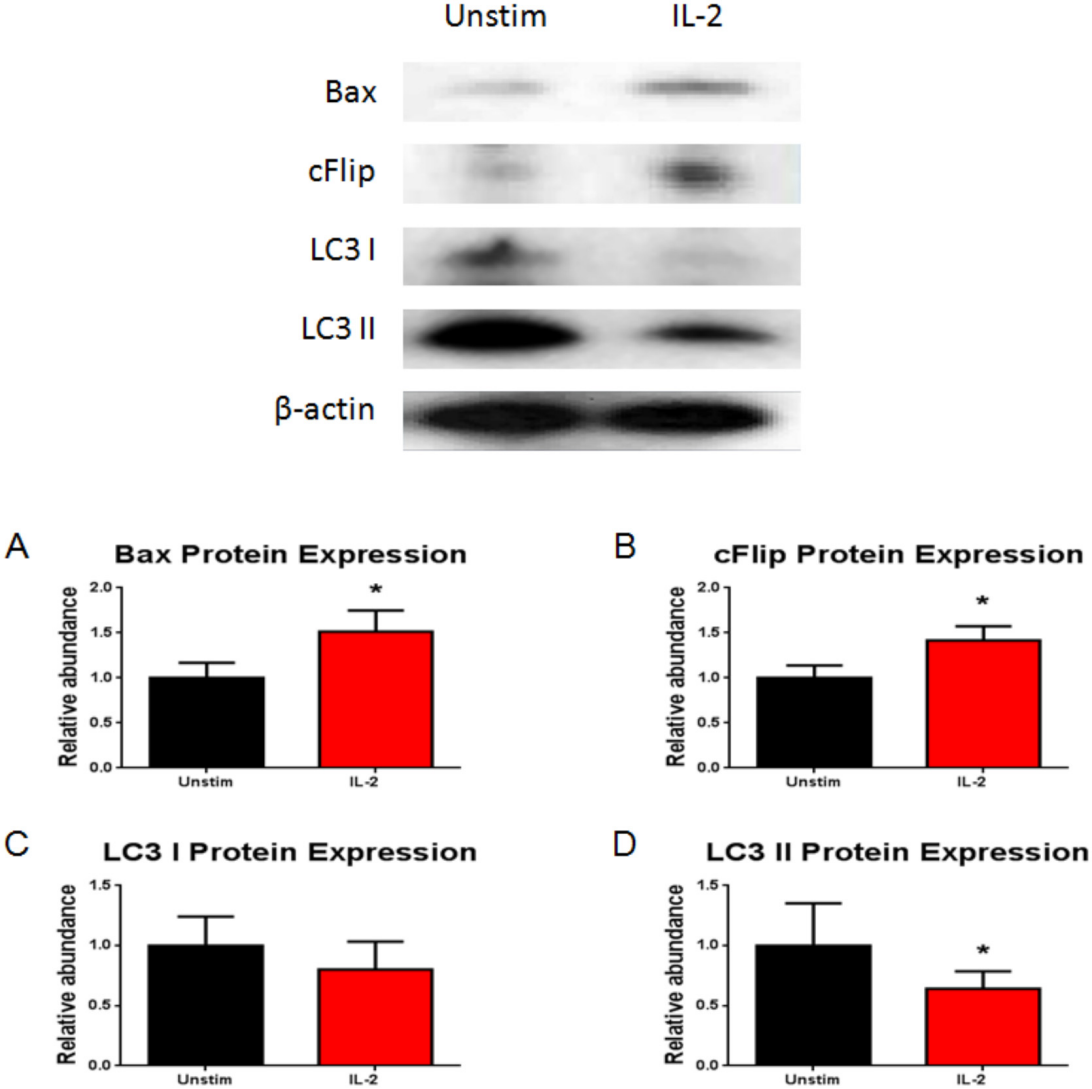
IL-2 activates the podocyte mitochondrial intrinsic pathway and inhibits podocyte autophagy. The induction of apoptosis was mediated by the intrinsic pathway as reflected by the increased expression of (A) Bax (P<0.05, unpaired t-test) and (B) cFLIP (P<0.01, unpaired t-test). IL-2 resulted in inhibition of podocyte autophagy with decreased protein expression for LC3 II (P<0.05). N = 4 for individual groups. Bar columns and error bars represent mean and standard deviation respectively.

Podocytes may exacerbate glomerular damage and lead to their own injury by the expression of receptors associated to pathways that induce pro-inflammatory molecules. Therefore, we wanted to evaluate whether activation of the IL-2R plays a role in inducing podocyte apoptosis. We showed by flow cytometry that IL-2 caused a significant increase in the number of cells with depolarized mitochondria and increased apoptosis (Fig 5A and 5B). The induction of apoptosis was mediated by the intrinsic pathway as shown by significant increases in both Bax and cFLIP protein expression (Fig 6A and 6B). Lastly, we demonstrated that incubation with IL-2 resulted in inhibition of podocyte autophagy as shown by decreased protein expression of autophagosome marker, LC3 II (Fig 6D).

### Effect of IL-2 on the Filtration Barrier Function of Podocytes

**Fig 7:**
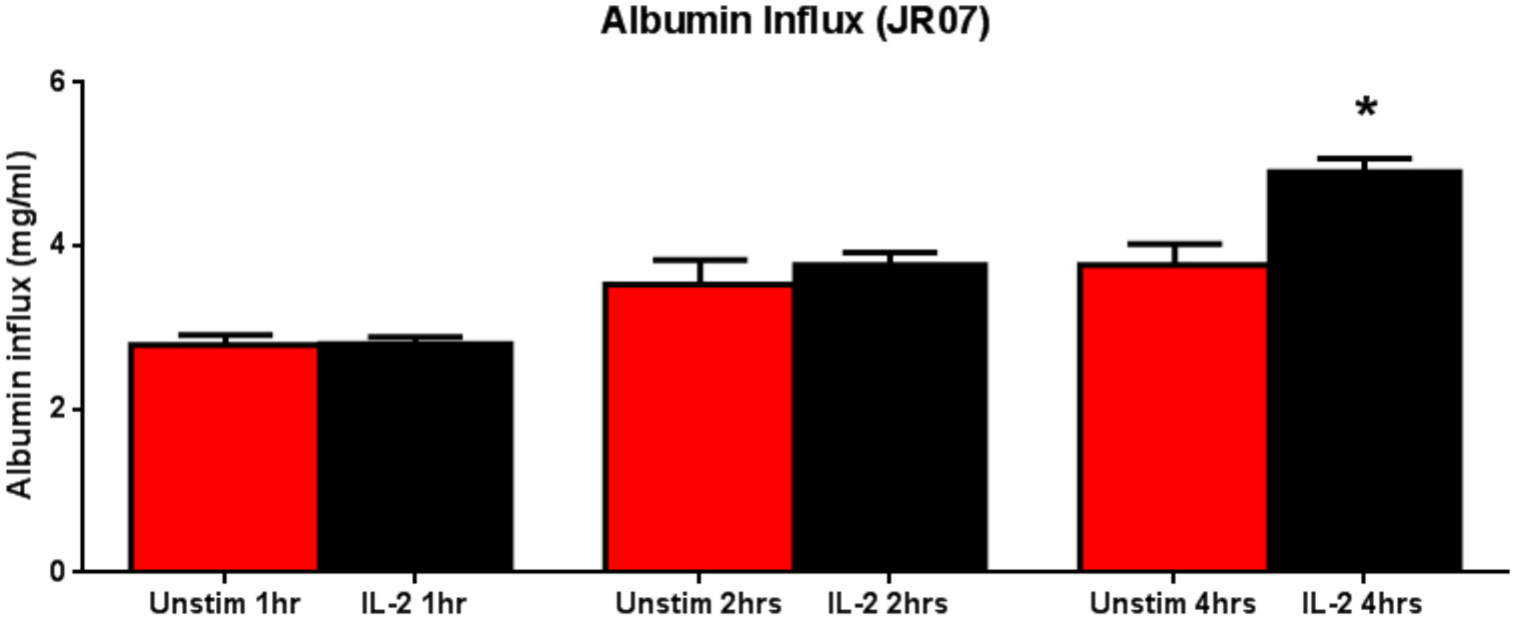
IL-2 increases podocyte paracellular albumin permeability. Murine podocytes (JR07) were seeded onto transwell filters (3μm pore, Corning) at (5–6 X 10^3^ cells/well) and allowed to differentiate for 10 days. Compared with the controls, IL-2 treatment resulted in a greater albumin influx across the podocyte monolayer (P<0.05, One-way ANOVA). N = 3 for individual groups. Bar columns and error bars represent mean and standard deviation respectively.

To assess the functional consequence of incubating the podocytes with IL-2, we examined the filtration barrier function of podocytes by using a paracellular permeability influx assay. Differentiated podocytes were incubated with IL-2 for 72 hours, and then subjected to albumin influx assay. As shown in Fig 7, compared with the controls, IL-2 treatment resulted in a greater albumin influx across the podocyte monolayer. These results indicate that the filtration barrier function of podocytes is impaired after exposure of the podocyte monolayer to IL-2.

## Discussion

Our study showed that murine podocytes express the IL-2R and that the receptor is activated with exposure to IL-2. The activation of the receptor is mediated by the JAK/STAT pathway. The data also showed that murine podocytes have reduced autophagy when stimulated with IL-2. In addition, IL-2 exposure increases protein expression of pro-apoptotic molecules and increases apoptosis in podocytes. The IL-2 induced apoptosis is mediated via the intrinsic (mitochondrial) pathway. Finally, we showed that a podocyte monolayer exposed to IL-2 has diminished filtration barrier function.

IL-2 is a cytokine involved in the growth and differentiation of T and B cells. It also plays an important role in proliferation of non-lymphoid cells [30, 31]. IL-2 signaling is mediated by a receptor complex (IL-2R) that consists of three subunits: IL-2Rα (CD-25), IL-2Rβ (CD-122) and the common γ chain (CD-132). There are three types of IL-2 receptor complexes: low, intermediate, and high affinity. The low affinity complex involves only the alpha chain and is not considered to be involved in signal transduction [32]. The intermediate affinity receptor involves both beta and gamma chains and are found to be expressed natural killer cells, macrophages, and resting T cells [32]. The high affinity receptor complex involves alpha, beta, and gamma chains and are found on activated T cells. The high affinity receptor complex is known to mediate most of the IL-2 effects in vivo (32). The IL-2R receptor signaling downstream is mediated by the activation of the tyrosine kinases JAK 1 and JAK 3 (Janus kinases 1 and 3), which are associated with the IL-2R β and γ subunits, respectively [33]. During activation, the tyrosine residues in the cytoplasmic domains of the IL-2R β and γ become phosphorylated, providing docking sites for the signaling proteins STAT 5 and to a lesser extent STAT 3 [34]. STAT’s themselves become phosphorylated, released into the cytoplasm and dimerized. The phosphorylated STAT dimer then translocates to the nucleus to regulate gene expression [35]. Activation of the JAK/STAT pathway plays a role in renal diseases such as diabetic nephropathy, where it results in activation of TGF-β and the subsequent development of glomerular sclerosis [36].

The expression of IL-2R is not restricted to lymphoid tissues. Functional IL-2R has also been reported in fibroblasts, endothelial cells, intestinal endothelial cells and tumors [37–40]. In kidney transplant rejection, the IL-2R has also been reported to be increased at the sites of inflammation, consistent with the known infiltration of the renal parenchyma by T-lymphocytes during acute rejection [41].

Podocyte injury plays a major role in INS [42, 43]. Chronic stimuli could affect the structural function of the podocytes and result in proteinuria [44]. The development of apoptosis plays an important role in podocyte depletion and glomerulosclerosis [45]. In diabetic nephropathy, high glucose concentration results in podocyte apoptosis [46]. The increased production of reactive oxygen species is the proposed mechanism for induction of podocyte apoptosis in this model [46]. The molecular mechanisms resulting in podocyte apoptosis in chronic progressive glomerular conditions remains poorly understood.

We evaluated conditionally immortalized murine podocytes for the presence of IL-2R mRNA by real time PCR. Our results demonstrated the presence of the three subunits of the IL-2R in differentiated podocytes by flow cytometry (Fig 1A). We also demonstrated that the alpha subunit was present by real time PCR and in podocyte protein extracts by Western blot (Fig 1B and 1C, respectively). Stimulation of murine podocytes with IL-2 induces the expression of the three subunits of the IL-2R (Fig 2). By Western blot we are demonstrating that murine podocytes express the high affinity receptor complex.

We also performed dose-kinetics to determine the most adequate concentration of IL-2 to be used in our experiments. The IL-2 concentration was found to be consistent with established concentrations used to induce vascular leak syndrome (VLS) in vivo [23, 47, 48]. We would like to note that at 100 ng/ml, this concentration is far above the plasma concentrations ever detected in vivo. However, IL-2R alpha is considered to be the private receptor for the IL-2 cytokine, thus limiting the possibility of having a non-specific interaction between IL-2 with other receptors lacking the IL-2R alpha subunit (32).

Signaling through the IL-2R in lymphocytes, results in the phosphorylation of several proteins including JAK 3 and STAT 5 [33]. Activation of the JAK-STAT pathway plays a role in renal diseases such as diabetic nephropathy, where it results in activation of TGF-β and the subsequent development of glomerular sclerosis [36]. Our results demonstrate that activation of the IL-2 receptor induces the JAK 3 and STAT 5 pathway (Figs 3 and 4). There was a decrease in podocyte nuclear phosphorylated Stat5 when the cells were incubated with IL-2. A potential explanation for this observation may be a result of the selected stimulation time points. Prior studies looking at phosphorylated Stat5 showed peak phosphorylation at 5 minutes [49, 50]. In spite this limitation, we demonstrated that IL-2 activates the Jak-Stat5 pathway in podocytes. Subsequently our goal was to evaluate the effect of activating the IL-2R in podocytes.

Podocytes may exacerbate glomerular damage and lead to their own injury by the expression of receptors associated to pathways that induce pro-inflammatory molecules. TGF-β is an example of a cytokine that plays a role in various cellular processes such as cell growth, differentiation, and apoptosis [26]. Several studies have shown that increased levels of TGF-β are found in injured kidneys of both experimental animals and in humans with chronic kidney disease [51, 52].

The role of TGF-β in inducing podocyte apoptosis was demonstrated by Schiffer et al. [53]. Apoptosis of podocytes was induced in TGF-β1 trans-genic mice by activation of p38 MAP kinase and caspase-3 [54]. Our results showed that IL-2 induced increased mitochondrial depolarization and podocyte apoptosis (Fig 5). This is a sensitive marker for cellular injury. The induction of apoptosis by IL-2 is mediated by activation of the mitochondrial intrinsic pathway as shown by the significant increase in pro-apoptotic markers Bax and cFLIP (Fig 6A and 6B, respectively). Our results demonstrate that activation of the podocyte IL-2R results in podocyte injury.

Autophagy plays an essential role in maintaining viability in terminally differentiated cells [24]. Podocytes have an increased basal level of autophagy and defective autophagy could play a role in facilitating injury as demonstrated in models of diabetic nephropathy [25]. Inhibition of autophagy could induce podocyte apoptosis by activation the pro-apoptotic pathway of the endoplasmic reticulum [26]. Our results showed that podocytes maintain an increased level of autophagy as reported in previous studies.

IL-2 stimulation resulted in decreased podocyte autophagy as reflected by the decreased expression of LC3 II (Fig 6D). This might be a contributing factor in the podocyte injury that we observed after incubation with this cytokine.

To assess the functional consequence of incubating podocytes with IL-2, we examined the filtration barrier function of podocytes by using a paracellular permeability influx assay [54]. As shown in Fig 7, compared with the controls, IL-2 treatment resulted in a greater albumin leakage across the podocyte monolayer. These results indicate that the filtration barrier function of podocytes is impaired after exposure to IL-2.

As current therapies to treat INS include steroids and calcineurin inhibitors, our results are significant. Glucocorticoids suppress IL-2 signaling in lymphocytes [55]. Experimental and clinical studies have shown that calcineurin inhibitors induce reversible inhibition of IL-2 production by lymphocytes [56]. Since both agents influence the structure and function of podocytes our results on the expression of the IL-2R support an additional mechanism for the direct effect of these agents in INS [57].

In summary, we have shown that murine podocytes express a functional IL-2R. When activated by the presence of IL-2, the IL-2R induces the upregulation of pro-apoptotic molecules and a decrease in the self-regulatory mechanism of autophagy. The combination of these two factors leads to increased podocyte apoptosis and a loss of the structural integrity of the podocyte filtration barrier. The results characterize the mechanism by which IL-2 causes podocyte injury. These findings support an important functional link between podocyte cell signaling and IL-2, a known target of current immunosuppressive therapy for nephrotic syndrome.
